# A Heavy Metal-Associated Protein (AcHMA1) from the Halophyte, *Atriplex canescens* (Pursh) Nutt., Confers Tolerance to Iron and Other Abiotic Stresses When Expressed in *Saccharomyces cerevisiae*

**DOI:** 10.3390/ijms150814891

**Published:** 2014-08-22

**Authors:** Xin-Hua Sun, Gang Yu, Jing-Tao Li, Pan Jia, Ji-Chao Zhang, Cheng-Guo Jia, Yan-Hua Zhang, Hong-Yu Pan

**Affiliations:** Plant Science, Jilin University, Changchun130062, Jilin, China; E-Mails: xinhua_sun@126.com (X.-H.S.); chrisyu_gang@126.com (G.Y.); lijingtao789@126.com (J.-T.L.); jiapanpan1994@126.com (P.J.); zhang_ji_chao@126.com (J.-C.Z.); jiacg@jlu.edu.cn (C.-G.J.)

**Keywords:** HMA (heavy metal-associated) domain, *Atriplex canescens*, Fe tolerance, abiotic stress, yeast expression

## Abstract

Many heavy metals are essential for metabolic processes, but are toxic at elevated levels. Metal tolerance proteins provide resistance to this toxicity. In this study, we identified and characterized a heavy metal-associated protein, AcHMA1, from the halophyte, *Atriplex canescens*. Sequence analysis has revealed that AcHMA1 contains two heavy metal binding domains. Treatments with metals (Fe, Cu, Ni, Cd or Pb), PEG6000 and NaHCO_3_ highly induced *AcHMA1* expression in *A. canescens*, whereas NaCl and low temperature decreased its expression. The role of AcHMA1 in metal stress tolerance was examined using a yeast expression system. Expression of the *AcHMA1* gene significantly increased the ability of yeast cells to adapt to and recover from exposure to excess iron. AcHMA1 expression also provided salt, alkaline, osmotic and oxidant stress tolerance in yeast cells. Finally, subcellular localization of an AcHMA1/GFP fusion protein expressed in tobacco cells showed that AcHMA1 was localized in the plasma membrane. Thus, our results suggest that *AcHMA1* encodes a membrane-localized metal tolerance protein that mediates the detoxification of iron in eukaryotes. Furthermore, AcHMA1 also participates in the response to abiotic stress.

## 1. Introduction

A number of heavy metals, including copper (Cu), iron (Fe), manganese (Mn), nickel (Ni) and zinc (Zn), are essential micronutrients required for a wide variety of plant physiological processes [1,2]. Copper, for example, functions as a cofactor in enzymatic catalysis, in the biochemistry of cellular respiration, antioxidant defense and iron metabolism in eukaryotes [3]. Iron is required for life-sustaining processes, from respiration to photosynthesis. Excess heavy metals, however, are toxic. They can inactivate biomolecules by blocking essential functional groups or by taking the place of other species of metal ions [4]. In addition, Cu, Fe and other metals can take part in reactions that generate reactive free radicals, superoxides and hydrogen peroxide. Free radicals react with water to generate the hydroxyl radical, which can damage cellular components, such as DNA, proteins, lipids and sugars [5,6]. In order to maintain metal homeostasis, plants have evolved diverse mechanisms to modulate the concentrations of metal ions, as well as to exclude non-essential forms by controlling their uptake, extrusion, chelation, distribution, storage and sequestration [7].

Proteins that transport heavy metals in microorganisms, mammals and plants share similarities across the kingdoms in their sequences and structures. The same heavy metal-associated domain (HMA, pfam00403.6) is characteristic of heavy metal transporters and detoxification proteins. The HMA exhibits a core amino acid sequence motif, M/L/IxCxxC, with two conserved cysteine residues that are involved in heavy metal binding [8,9]. Some HMA domain-containing proteins from animals, yeast, bacteria and plant species specifically bind copper and are involved in copper homeostasis [8,10]. The HMA domain also showed binding activity with other heavy metals, such as nickel or zinc [9,11]. MerP, a bacterial carrier for mercury ions, could sequesters Hg^2+^ ions in the periplasmic space [12]. Analysis of several HMA-containing proteins from a variety of different organisms has demonstrated that they play an essential role in heavy metal transport and homeostasis [11,13].

*Saccharomyces cerevisiae* is a model organism for the study of fundamental cellular processes, including the uptake, metabolism and homeostatic control of mineral nutrients and trace elements [14]. Riger and his colleagues used a yeast system to identify the mutagenic effect of metals upon human p53 and to evaluate general heavy metal toxicity in eukaryotes [15]. In addition, yeast is an excellent organism for studying the mechanisms underlying stress tolerance [16,17,18,19]. Expression of GhBCP1 and GhBCP4 in yeast significantly increased the cell growth rate under Cu^2+^, Zn^2+^ and high salinity stresses [20].

Four-wing saltbush (*Atriplex canescens*) is a halophyte that shows high tolerance to salinity, drought, heavy metals and temperature. *A. canescens* is a xerophyte, naturally found in deserts and used for arid zone restoration projects [21]. Previously, in order to better understand the mechanisms of stress tolerance in *A. canescens*, a full-length cDNA library was generated from leaf and root tissues that had been conditioned to 400 mM NaCl. The library provided 343 high-quality Expressed Sequence Tags (ESTs) [22]. Here, the gene for the HMA domain containing protein AcHMA1 was isolated from the cDNA library. Quantitative real-time RT-PCR was performed to reveal the expression pattern of *AcHMA1* under different abiotic stresses. To begin to elucidate its role, we examined the tolerance of AcHMA1 transgenic yeast to Cu^2+^, Fe^2+^, Fe^3+^, Zn^2+^, Pb^2+^, Cd^2+^, Ni^2+^, Mn^2+^ and Co^2+^. This transgenic yeast harboring AcHMA1 was also used to gain insight into the role of AcHMA1 in salt, alkaline, osmotic and oxidant stress. Subcellular localization analysis of AcHMA1 was investigated by the transient expression of an AcHMA1-GFP fusion protein in *Nicotiana benthamiana* Domin.

## 2. Results and Discussion

### 2.1. Sequence Characterization and Deduced Amino Acid Sequence Comparison

**Figure 1:**
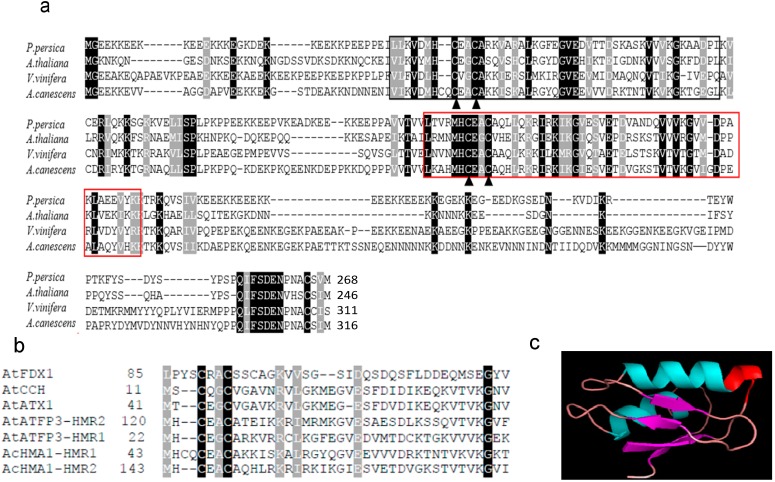
(**a**) Structure-based sequence alignment between AcHMA1 and the homolog from different organisms. The proteins shown are from *P. persica*, *A. thaliana*, *V. vinifera* and *A. canescens*; a black background indicates conserved residues; a brown background indicates residues identified to be more than 80% conserved. The conserved heavy metal-associated domains are marked by a black frame (HMRI) and a red frame (HMR2); the key residues (two cysteine residues) important for metal ions binding and transfer are marked by triangles; (**b**) Alignment with proteins containing CXXC (heavy metal binding core motif) domains from *A. thaliana*. The predicted AcHMA1 protein contains two CXXC motifs, the first of which begins at amino acid 47 and the second of which begins at amino acid 155. Farnesylated protein 3 (AtATFP3); copper transport protein (AtATX1); copper transport protein (AtCCH); ferredoxin (AtFDX1); (**c**) Predicted 3D structure model of the AcHMA1 protein. The model of the AcHMA1 protein was created by the Phyre server. Helices, strands and coils are colored cyan, purple and violet, respectively. The putative metal binding sites are highlighted in red.

A 1270-bp full-length cDNA copy of the *AcHMA1* (KF863910) gene was cloned from an *A. canescens* cDNA library. The putative ORF encodes a polypeptide of 316 amino acid residues containing 13.6% lysine with a predicted molecular mass of 35.5 kDa, an isoelectric point of pH 8.6 and a calculated isoelectric point of pH 8.6. It contains two heavy metal binding core motif (CXXC) [23]. These are thought to be binding sites for transition metal ions [24]. The first site (HMRI) begins at amino acid 38, and the second (HMR2) begins at amino acid 147 (Figure 1a).

We utilized BLASTP analysis to search for AcHMA1 homologs. AcHMA1 was found to be similar to a hypothetical protein from *Vitis vinifera* L. (Figure 1a). Searching for homologs of the CXXC metal-binding domain in proteins involved with heavy metal homeostasis and phylogenetic analysis of these proteins revealed that the amino acid sequences of these proteins are not conserved, except for the key cysteine residues (Figure S1). The CXXC metal-binding domain was found in proteins involved with heavy metal homeostasis in *A. thaliana*, including farnesylated protein 3, two copper transport proteins and ferredoxin. The amino acid sequences of these domains were not conserved, except for the key cysteine residues (Figure 1b). The two heavy metal binding regions were found to take on a ferredoxin-like αβαββαβ secondary structure, which was also predicted from the three-dimensional structure of the AcHMA1 protein (Figure 1c).

### 2.2. Differential Regulation of AcHMA1 Expression in Response to Various Abiotic Stresses

**Figure 2:**
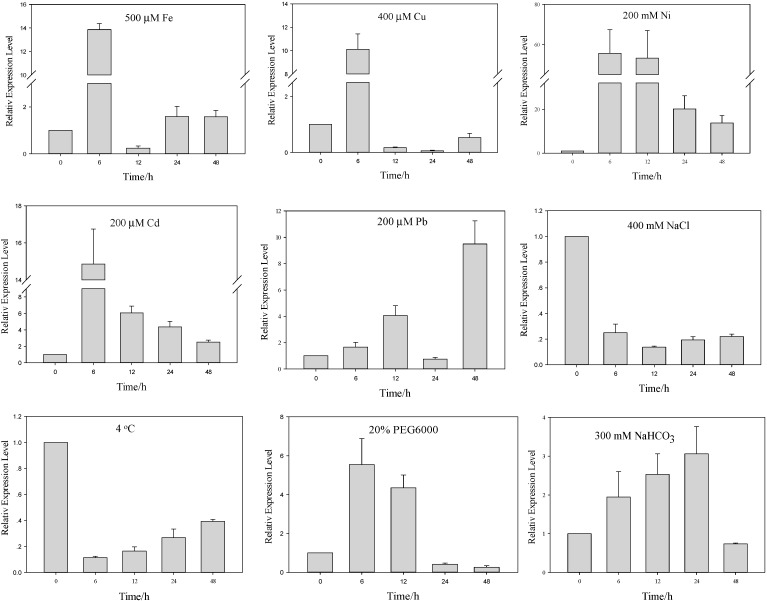
Quantitative RT-PCR validation of *AcHMA1* under 500 µM Fe, 400 µM Cu, 200 µM Ni, Cd and Pb, 400 mM NaCl, 300 mM NaCHO_3_, 20% PEG6000 and low temperature treatments. EF1α was used as the internal control. Relative expression levels of AcHMA1 in stress treatments were plotted as the relative expression fold of the non-treated seeding (0 h) for 6, 12, 24 and 48 h. Error bars: standard errors.

HMA domain-containing proteins participate in metal ion metabolism and bind Cd, Hg, Ni, Pb, Fe and Cu and can be induced by these metals; this capacity for metal induction was tested in *A. canescens*. Quantitative RT-PCR was performed using the total RNA extracted from three-month-old plants subjected to various stress treatments at different time intervals as described in the Experimental Section. The time-dependent expression profiling revealed different patterns of transcript regulation for *AcHMA1* in response to various metals and other abiotic stresses (Figure 2). Seedlings treated with Fe displayed a 13-fold upregulation of *AcHMA1* at 6 h post stimulation, but dropped to 0.2-fold at 12 h, then subsequently increased again to 1.5-fold at 24 h. Cu stress caused *AcHMA1* expression to reach its highest level at 6 h and then to suddenly reduce to the pre-induction level. Treatment with Ni, Pb and Cd also led to a significant increase in *AcHMA1* transcript levels. On exposure to Ni stress, the *AcHMA1* transcript level reached its highest peak in the first 6 h and maintained this level up to 12 h, a 50–55-fold increase in *AcHMA1* expression levels relative to EF1α, then reduced, but was still higher than the pre-induction level. Under Pb treatment, there were two peaks: the transcript accumulation was induced at 6 h and reached a high level at 12 h; then, transcript levels declined at 24 h, but peaked at an even higher level at 48 h, suggesting a feedback adjustment. The transcript level of *AcHMA1* in response to Cd treatment reached its highest level at 6 h and subsequently reduced slowly during the following time.

A different regulation pattern was observed in the case of salt and cold stress, *AcHMA1* expression was strongly downregulated under NaCl stress and cold stress, with transcript levels showing a strong decrease at all points studied. For osmotic stress, strong induction was observed with a maximum 5.5-fold increase at 6 h, decreasing to 4.3-fold thereafter. The effect of alkali exposure was less pronounced than that of PEG6000. On exposure to alkaline stress, the *AcHMA1* transcript levels increased in the first 6 h and continued to increase up to 12 h, reaching its highest level at 24 h post-stimulation. All of these results showed that *AcHMA1* expression regulation is specific to each particular inducer.

### 2.3. Overexpression of AcHMA1 Improves Iron Tolerance in Yeast Cells

**Figure 3:**
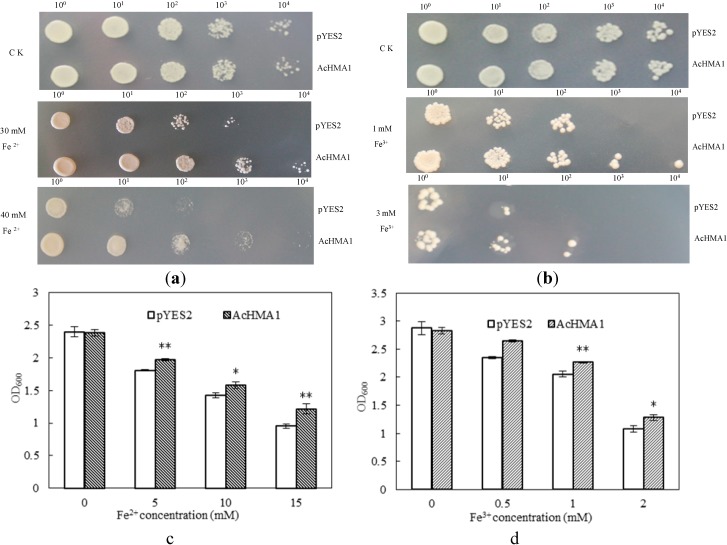
Fe tolerance of *AcHMA1*-transformed yeast. (**a**,**b**) Yeast cells transformed with vector pYES2 or with *AcHMA1*-pYES2 were grown in Synthetic complete medium dropout uranium (SC-U) for 24 h, and their OD_600_ were adjusted with SC-U to 1, 0.1, 0.01, 0.001, 0.0001, two microliters of diluted cultures were spotted onto medium supplementing excess Fe^2+^ or Fe^3+^; (**c**,**d**) Growth of yeast cells expressing *AcHMA1* and the control cell in the medium containing different concentrations of Fe^2+^ or Fe^3+^. The yeast transformants were grown in medium containing 2% galactose for 24 h at 28 °C, then adjusted OD_600_ = 2 in 1 mL of medium for experiments. The cells were in 5 mL cultures containing different concentrations of Fe at 28 °C for 24 h with shaking. Cell densities were measured (OD_600_) after each treatment. The yeast cell incubated in medium containing 2% galactose was measured after 24 h as the control. The data are expressed as the mean ± SE of three replicates; * and ** indicate significant levels at 5% and 1%, respectively. Yeast cells transformed with vector pYES2 were used as control.

To characterize the heavy metal stress tolerance conferred by the expression of *AcHMA1*, two yeast lines were constructed. One was transformed with an empty vector pYES2 as the control (WT), and the other was transformed with pYES2-*AcHMA1*. Growth of WT and *AcHMA1*-transfromed yeast cells showed no apparent difference on basal medium (Figure 3). While the amount of available iron had a notable effect on the growth of the cultures, above a certain level, the iron was found to be inhibitory to growth. However, both spot assays and growth curves showed that the *AcHMA1*-transfromants had significantly better survival rates than wild yeast in the presence of excess iron (Fe^2+^ or Fe^3+^). For the spotting assay, when yeast cultures were diluted to a density of 0.01 with 30 mM Fe^2+^ (Figure 3a), *AcHMA1*-transformants grew better than the WT. Moreover, as the Fe^2+^ concentration increased to 40 mM (Figure 3a), the growth of the WT fell below levels of growth limited by Fe^3+^ at 1 mM (Figure 3b). The yeast cells expressing *AcHMA1* grew well even when the cultures were diluted to 0.0001; however, the WT could not survive even when the cultures were only diluted to 0.001. These results indicate that yeast expressing *AcHMA*1 were more tolerant to iron stress than was the WT. The effects were most evident from the growth curves (Figure 3c,d). To determine whether expression of *AcHMA1* increased tolerance to other heavy metals, we cultivated the two lines of yeast cells on medium supplemented with 5 mM Cu^2+^ or 10 mM Cu^2+^, 10 mM Ni^2+^, 1.5 mM Pb^2+^, 5 mM Cd^2+^, 10 mM Mn^2+^, 15 mM Co^2+^ and 15 mM Zn^2+^. No significant differences were noted comparing the growth of WT with that of yeast cells transformed with pYES2-*AcHMA1* (Figure S2).

### 2.4. Response of the Transgenic Yeast Cells Expressing AcHMA1 to Abiotic Stresses

**Figure 4:**
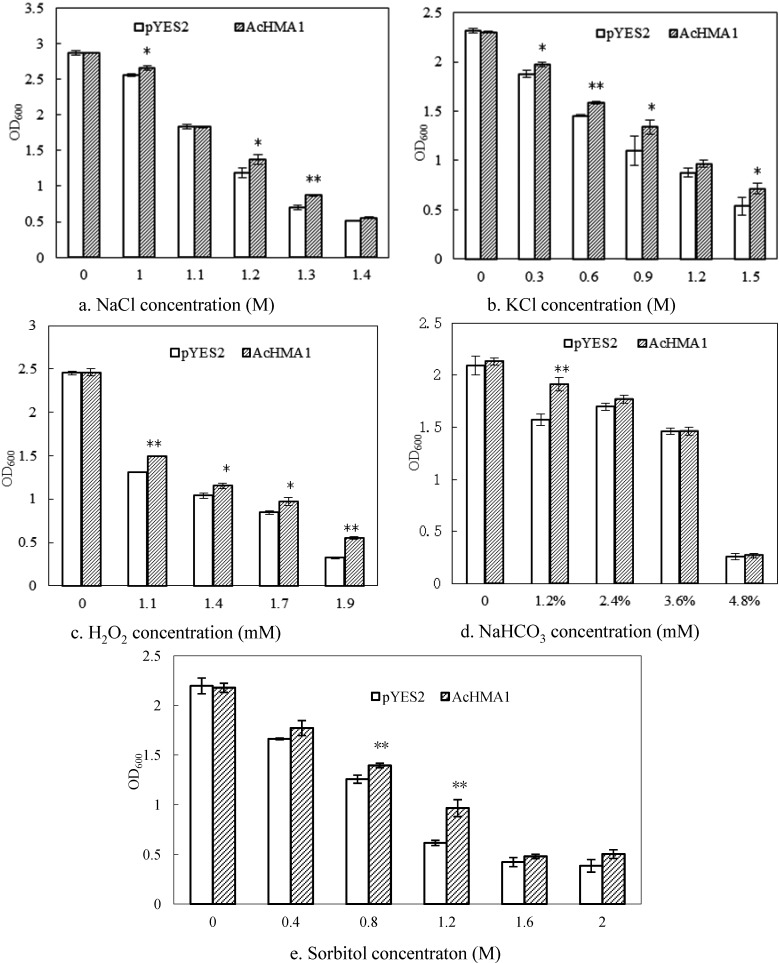
Stress response analysis of *AcHMA1* overexpressing yeast cells. Growth of yeast cells expressing *AcHMA1* and the pYES2 in the medium containing 1.0, 1.1, 1.2, 1.3, 1.4 M NaCl (**a**); 0.3, 0.6, 0.9, 1.2, 1.5 M KCl (**b**); or 1.1, 1.4, 1.7, 1.9 mM H_2_O_2_ (**c**). For osmotic and alkali stress, the yeast cells were cultured in the medium containing 1.2%, 2.4%, 3.6%, 4.8% NaHCO_3_ (**d**); or 0.4, 0.8, 1.2, 1.2, 1.6, 2.0 M sorbitol (**e**). * and ** indicate significant levels at 5% and 1%, respectively.

To determine if *AcHMA1* gene expression conferred other abiotic stress tolerance in transgenic yeast, the two yeast lines were treated with different abiotic stressors. No difference in growth levels were noted between the *AcHMA1* transgenic and control yeast under normal culture conditions (Figure 4). However, *AcHMA1*-expressing yeast cells exhibited better growth than did the control in the presence of salt (NaCl, KCl), osmotic (sorbitol) and oxidant (H_2_O_2_) stress conditions (Figure 4), indicating that *AcHMA1* transformed yeast cells possessed increased tolerance to these abiotic stressors.

### 2.5. AcHMA1 Protein Localization to the Plasma Membrane of Plant Cells

**Figure 5:**
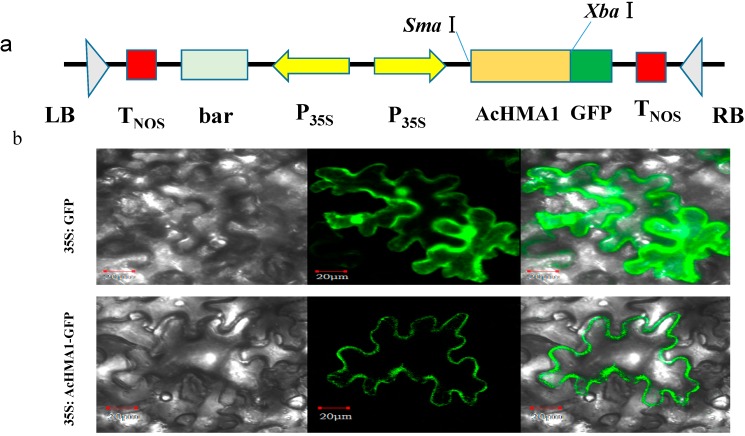
Subcellular localization of AcHMA1-GFP. (**a**) Schematic representation of constructs used for transformation and subcellular localization of the AcHMA1protein; (**b**) Subcellular localization assay of the AcHMA1 protein. Images show *N. benthamian*a epidermal cells expressing GFP (control, **upper lane**) or the AcHMA1:GFP fusion protein (**bottom lane**); bright-field illumination (**left**), followed by fluorescent-field illumination was used to examine GFP fluorescence (**middle**) and confocal microscopy (**right**) for an overlay of bright and fluorescent illumination. Bars = 20 µm.

To examine the subcellular distribution of the AcHMA1 protein, we fused GFP to AcHMA1 (Figure 5a). Confocal imaging showed that the AcHMA1-GFP fusion protein localized exclusively in the plasma membrane of tobacco epidermal cells in the transient expression assay (Figure 5b). In contrast, free GFP was also found in both the nucleus and cytosol.

## 3. Experimental Section

### 3.1. Cloning and Sequence Analysis of the AcHMA1 Gene from *A. canescens*

The full-length cDNA sequence of *AcHMA1* was cloned from an *A. canescens* cDNA library after EST sequencing and analysis. The functional annotation of ESTs was performed using the BLASTX program [25]. The EST representing *AcHMA1* was identified, and its sequence was confirmed. The putative heavy metal-associated (HMA) domain was characterized by Pfam [26] and by the BLASTP servers at NCBI [27]. DNAsis software was used to predict AcHMA1’s secondary structure. The sequence alignment of AcHMA1 with other heavy metal-associated proteins from plants, including *Prunus persica*, *Arabidopsis thaliana* and *V. vinifera*, was performed using CLUSTALX1.81, and a phylogenetic tree was constructed using the neighbor-joining method with MEGA5. The three-dimensional (3D) structure of AcHMA1 (amino acid residues 23–116) was generated by protein modeling in the Phyre server [28], using copper transport protein ATOX1 (GenBank Number CR4569011; PDB Code O00244) as the template.

### 3.2. Plant Abiotic Stress Treatments to Study AcHMA1 Gene Regulation

*A. canescens* seedlings were grown in Hoagland solution in a greenhouse under conditions of 60%–70% relative humidity, 14 h of light and an average temperature of 23 °C. For iron (Fe), copper (Cu), cadmium (Cd), nickel (Ni) and lead (Pb) treatments, 3-month-old seedlings of *A. canescens* were treated with ferric sulfate at 500 µM, copper sulfate at 400 µM, cadmium chloride at 200 µM, lead nitrate at 200 µM or nickel chloride at 200 µM [11,29]. Tissue was collected after 0, 6, 12, 24 and 48 h. Plants were exposed to salt stress by adding 400 mM sodium chloride to the Hoagland solution. For cold treatment, plants were transferred to a growth chamber maintained at 4 °C. Osmotic and alkaline stress was implemented by supplementing the Hoagland solution with 20% PEG6000 (*v/v*) or 300 mM sodium bicarbonate [30]. All samples were harvested, snap frozen and stored at −80°C until needed.

### 3.3. RNA Isolation and Quantitative RT-PCR Assay

Total RNA was extracted from hydroponically grown *A. canescens* plants using TRIzol reagent (Invitrogen, Carlsbad, CA, USA). Two micrograms of total RNA were used for reverse transcription with the SuperScript First-Strand Synthesis System kit (Invitrogen). The cDNA was 10-fold diluted, and quantitative expression assays were performed using the TaKaRa SYBR^®^Green Reagent kit with the 7500 real-time PCR detection system according to the manufacturer’s protocol (TaKaRa, Dalian, China). qRT-PCR conditions used were: 30 s at 95 °C followed by 40 cycles of 5 s at 95 °C, 34 s at 60 °C, 15 s at 95 °C, 60 s at 60 °C and 15 s at 95 °C. qRT-PCR experiments were replicated at least three times. The relative quantification method (2^−ΔΔ*C*t^) was used to evaluate quantitative variation between replicates [31]. The data were normalized against the elongation factor 1-alpha (EF1α) gene. The primer pairs used for qRT-PCR are listed in Table S1.

### 3.4. Yeast Cultures and Transformation

*Saccharomyces cerevisiae* strain INVSc1 (*MAT*a *his3*D*1 leu2 trp1-289 ura3-62*) was used in this study (Invitrogen Co., Shanghai, China). Synthetic complete (SC) medium lacking specific nutrients was employed for the selection and maintenance of transformed yeast [32]. The full-length ORF of *AcHMA1* was obtained by conventional PCR utilizing the *Bam*HI forward primer 5'-CGCGGATCCATGGGAGAGGAAAAGAAAGAG-3' and the *Xho*I reverse primer 5'-CCGCTCGAGTTACATAATAGAACAAGCAT-3', and the restriction digested product was inserted into yeast expression vector pYES2 (Invitrogen Co., Shanghai, China). The vector was then introduced into yeast strain INVSc1 using a lithium acetate method [33]. Two percent (*w*/*v*) galactose was added into the growth medium (SC medium with or without 2% agar) to induce the expression of *AcHMA1* under the control of the GAL1 promoter in the pYES2 plasmid [34].

### 3.5. Stress Tolerance Analysis

For the stress tolerance assay, yeast cells containing the vectors pYES2 (control) or pYES2-*AcHMA1* were grown in SC-U (without uracil) medium with shaking (200 rpm) at 28 °C for 24 h, and their OD_600_ was adjusted with SC-U to 1, 0.1, 0.01, 0.001, 0.0001, gradually. Two microliters of each diluted culture were spotted onto different SC-U plates containing 30 or 40 mM Fe^2+^ or 1 or 3 mM Fe^3+^. The plates were incubated at 28 °C for 4 days and then photographed. For yeast growth curves, yeast cells containing pYES2-*AcHMA1* or pYES2 were inoculated in SC-U medium with 2% glucose and grown for 24 h at 28 °C. The overnight cultures were centrifuged and adjusted to a final OD_600_ of 0.2 in 5 mL induction medium (SC-U medium with 2% galactose) containing different concentrations of stress agents: 5, 10, 15 mM Fe^2+^; 0.5, 1.0, 2.0 mM Fe^3+^; 1.0, 1.1, 1.2, 1.3, 1.4 M NaCl; 0.3, 0.6, 0.9, 1.2, 1.5 M KCl; or 1.1, 1.4, 1.7, 1.9 mM H_2_O_2_. Cultures were incubated at 28 °C with shaking 200 rpm for 24 h. For the osmotic stress test, the yeast cells were cultured in 0.4, 0.8, 1.2, 1.2, 1.6, 2.0 M sorbitol. For the alkali stress test, the cells were cultured in 0, 1.2%, 2.4%, 3.6%, 4.8% NaHCO_3_ (*m/v*) at 28 °C. Both of these were under the same conditions as described above. Cell density was measured (OD_600_) after the indicated treatment. The yeast cell transformed with pYES2 was used as the control with the identical treatments.

### 3.6. Subcellular Localization of AcHMA1 in N. benthamiana

The subcellular localization of AcHMA1 was determined using the *AcHMA1:GFP* fusion driven by a CaMV35S promoter. The *AcHMA1* ORF without a stop codon was amplified using primers (F: 5'-CGGAATTCATGTGTGGAGGTGCTGTAATTTCCG-3'; R: 5'-CCGCTCGAGTTAGAAGCCTCCTCCGGGGAAGTC-3'), digested with *Eco*RI and *Xho*I, then inserted into the binary vector, pCHF3000. The resulting construct was introduced into *Agrobacterium tumefaciens* EHA105 and transiently expressed in 4-week-old *N. benthamiana* as previously described [35]. The construct with GFP alone was used as the control.

### 3.7. Statistical Analysis

Statistical analysis was performed using the statistical software package SPSS 19.0 (SPSS Science, Chicago, IL, USA). A two-tailed *t*-test was conducted to analyze significant differences between the yeast cells transformed with vector pYES2 and pYES2-*AcHMA1* at 5% and 1% levels of probability. The experiments were repeated three times. The data is expressed as the mean ± SE.

### 3.8. Accession Numbers

The GenBank accession numbers of the mentioned sequences are: *Prunus persica* (L.) Batsch (EMJ13012.1), *A. thaliana* (AEE73885.1), *V. vinifera* (CAN62004.1) and *A. canescens* (KF863910.1) for alignment between AcHMA1 and homologues. *A. thaliana*: farnesylated protein 3 (AtATFP3), AAD09507; copper transport protein (AtATX1), Q94BT9.1; copper transport protein (AtCCH), AAC33510.1; ferredoxin (AtFDX1), AEE28669.1, for alignment of the CXXC-type metal-binding domains.

### 3.9. Discussion

The heavy metal-associated motif is a conserved domain of approximately 30 amino acid residues found in a number of proteins that transport or detoxify heavy metals. Examples of those proteins include the CPx-type heavy metal ATPases and copper chaperones. The domain contains two cysteine residues that are important in the binding and transfer of metal ions of copper, cadmium, cobalt and zinc [24]. The importance of these proteins in metal homeostasis is becoming clearer as a number of metal transporters have been identified as chelators for metal tolerance and for the mobilization and translocation of heavy metals into and within plants [36,37]. In this study, we characterized *AcHMA1* from *A. canescens* as encoding a heavy metal-associated protein. Quantitative RT-PCR demonstrated that *AcHMA1* was induced by various heavy metals: Fe, Cu, Ni, Cd and Pb (Figure 2). We found that the gene product provides tolerance to excess iron levels when expressed in yeast (Figure 3). Sequence analyses showed that *AcHMA1* encodes two HMA regions (Figure 1a) that show strong similarity to the motifs found in the copper chaperone, ATX1, and in AtATFP3 [10,38]. AtCCH, a functional homolog of ATX1, shares high sequence homology with ATX1 and is essential for copper homeostasis [39]. Both of them contain an HMA motif, which is repeated in AtATFP3. These metal-binding proteins, however, except for the core sequence of the HMA domain, do not bear other discernable sequence similarities to the AcHMA1 protein. According to Baxter *et al.*, 2003 [40], higher plants have evolved with a relatively high number of proteins containing the HMA domain in their genomes. These proteins have diversified toward a range of specific metals. Only the key residues that participate in metal binding and transport are likely to be conserved during this divergence. The deduced secondary structure prediction of AcHMA1 shows the ferredoxin-like αβαββαβ fold (Figure 1c). This is characteristic of many copper chaperones and chaperone-like proteins [41], such as the copper-binding protein, HAHI. HAHI functions as an intracellular copper chaperone mediating copper homeostasis and antioxidant defense in eukaryotic cells [8].

Taken together, the results of these analyses support the premise that AcHMA1 may act as a metal transporter involved in metal metabolism. To gain further insight into the function of AcHMA1, we used a yeast expression system to generate a transgenic *S. cerevisiae* strain that produces the *AcHMA1* gene product. Expression of the AcHMA1 protein by that strain appeared to provide tolerance to excess iron levels in the transgenic yeast (Figure 3). Metal tolerance assays showed that the gene in yeast cells provided tolerance specific to iron, with no effect seen with Cu^2+^, Ni^2+^ , Pb^2+^, Cd^2+^, Co^2+^, Mn^2+^ or Zn^2+^. qRT-PCR analyses indicated that *AcHMA1* transcripts in *Atroplex* were induced not only by Fe and Cu, but also by Ni, Cd and Pb. Previous research has shown that the HMA domain binds to and transfers metal ions, such as copper, cadmium, cobalt, zinc or iron [41], so most members of this family are metal responsive genes. Transcripts of CdI19, a metal binding protein from *Arabidopsis*, were induced not only by Cd, but also by Hg, Fe and Cu [11,13]. In *S. cerevisiae*, ATX1 is part of a pathway that links copper transport to iron uptake at the cell surface [38,42,43,44]. ATX1 binds free copper in the cytoplasm and delivers it to a copper transporter in the membrane of a post-Golgi vesicle [23]. Ultimately, copper becomes incorporated into a complex capable of reducing iron outside the cell [43]. We have shown that expression of *AcHMA1* improves iron tolerance in yeast cells. It is possible that the AcHMA1 protein may be part of such a copper transport pathway. A homologous copper transport pathway has been identified in *Arabidopsis*. *CCH* expression can restore high affinity iron uptake to *ATX1*-deficient yeast [39].

Metal transporter proteins are found in locations, such as the tonoplast and plasma membranes [36,37]. Exporting excess metal from cells via a transporter embedded in the plasma membrane or storing excess metal in vacuoles filled by vacuolar localized transporters are possible mechanisms for metal tolerance. Subcellular localization of the AcHMA1/GFP fusion protein showed that AcHMA1 localizes to the plasma membrane of tobacco cells (Figure 5), consistent with the metal transporter AcHMA1 function in heavy metal transporting.

One mechanism of heavy metal toxicity is the production of injurious reactive oxygen species (ROS) via the Fenton reaction [45]. ROS, such as O_2_^−^ or H_2_O_2_, may lead to the generalized oxidative modification of proteins, membrane lipids or DNA. There is an overlap between systems controlling metal-ion homeostasis and controlling oxygen radical metabolism [46,47]. In yeast, the copper chaperone, ATX1, contributes to both the maintenance of copper homeostasis and to the detoxification of the superoxide anion [48]. Similarly, *CCH*, the *Arabidopsis* homolog of ATX1, is involved in both the detoxification of active oxygen and the delivery of copper [39,49]. To determine whether the *AcHMA1* gene product is involved in oxidative stress, growth rates were compared between *AcHMA1*-expressing yeast cells and the yeast transformed with the empty pYES2 vector control. The results show that *AcHMA1*-transfromed yeast cells grew better than control cells under H_2_O_2_ stress conditions (Figure 4). Barth *et al.* [50] reported that HIPP26 from *A. thaliana* is involved in plants’ response to abiotic stresses, because its heavy metal-associated domain could interact with the drought stress-related zinc finger transcription factor, ATHB29. A mechanism based on the interaction of a metal bound to an HMA domain with a regulatory factor is already known from bacterial systems [51,52]. *AcHMA1* expression was strongly regulated by NaCl, cold, PEG6000 and NaHCO_3_. Moreover, *AcHMA1*-expressing yeast cells exhibited better growth than controls in the presence of salinity (NaCl, KCl), osmotic (sorbitol) and alkaline stress conditions (Figure 4), which indicates that *AcHMA1* may participate in the stress tolerance of plants by an interaction with stress-associated regulatory factors.

## 4. Conclusions

Overall, the data presented in this report suggest that AcHMA1 appears to be a plasma membrane-localized metal transporter that improves Fe tolerance and resistance to abiotic stress. In the transgenic yeast cells, the heavy metal binding protein improves resistance to salinity, oxidant, osmotic stress and alkali. qRT-PCR demonstrated that *AcHMA1* was induced by various heavy metals (Fe, Cu, Ni, Cd and Pb), as well as salinity, cold, osmotic stress and alkali; thus indicating that AcHMA1 may not only function as a metal transporter, but also, it may mediate plant response to abiotic stress. However, the exact mechanism by which AcHMA1 provides tolerance to multiple stresses is not known yet, and further research should focus on the role of AcHMA1 in plants under abiotic stress challenging conditions.

## Supplementary Files

Supplementary File 1
