# Supplementary material for: A Heavy Metal-Associated Protein (AcHMA1) from the Halophyte, Atriplex canescens (Pursh) Nutt., Confers Tolerance to Iron and Other Abiotic Stresses When Expressed in Saccharomyces cerevisiae
Source: Int J Mol Sci. 2014 Aug 22;15(8):14891–906. doi: 10.3390/ijms150814891 (PMC4159888; doi:10.3390/ijms150814891)
Supplement: Supplementary File 1 [file ijms-15-14891-s001.pdf]

Supplementary Information

Figure S1. Phylogenetic analysis of the heavy metal associated proteins from different plants. The unrooted Neighbor-Joining tree was constructed employing the MEGA4.0 software. Bootstrap values (percentages) higher than 50% (1000 replicates) are given at nodes. Branch length in the tree is proportional to the numbers of nucleotide substitutions as measured by the scale bar (1% sequence divergence). The AcHMAI described in this study is marked with triangle.

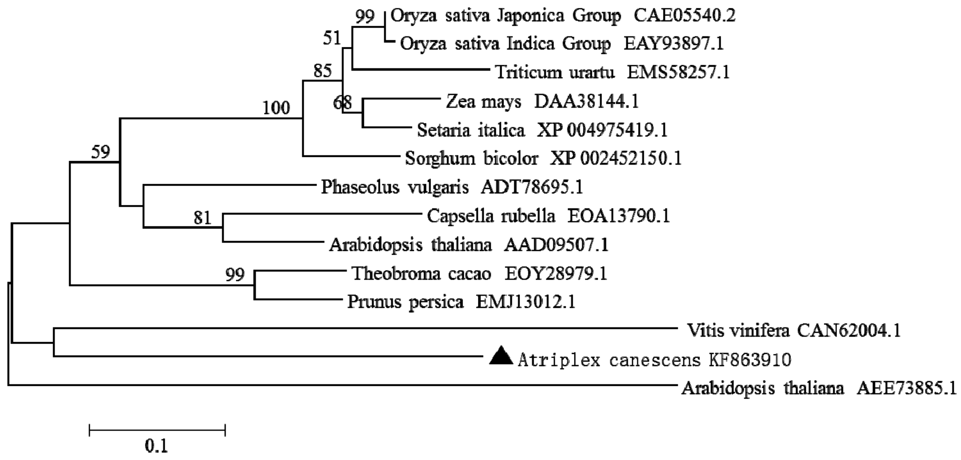

Figure S2. Metal stress response analysis of *AcHMA1*-overexpressing yeast cells. Growth of yeast cells expressing *AcHMA1* and the pYES2 in the medium supplemented with 1.5 mM Pb^{2+}, 5 mM Cd^{2+}, 10 mM Ni^{2+}, 10 mM Mn^{2+}, 15 mM Co^{2+}, 15 mM Zn^{2+}, 5 mM Cu^{2+} and 10 mM Cu^{2+}.

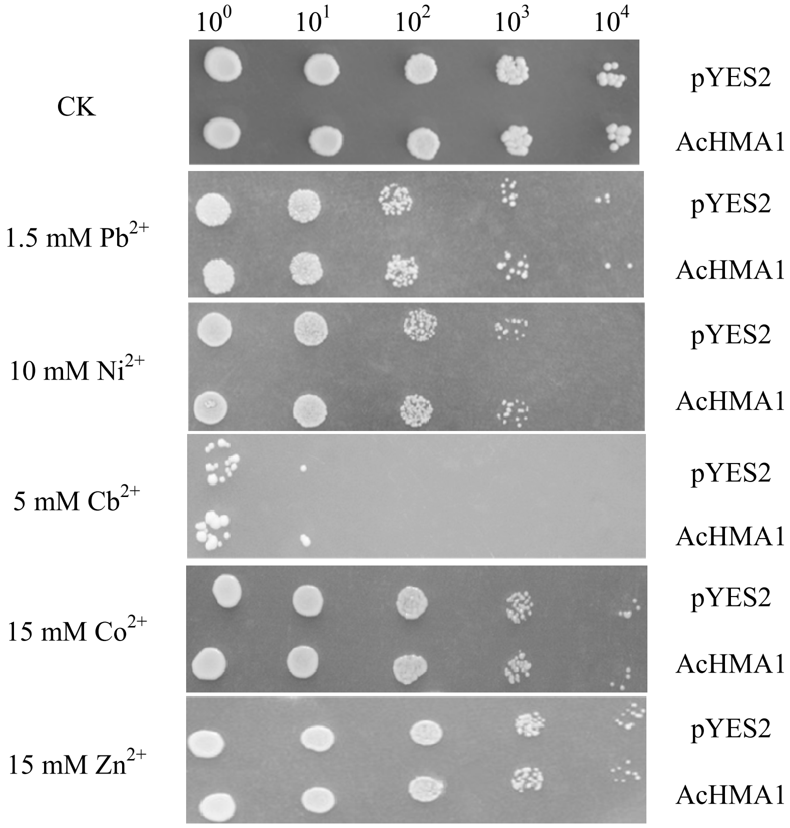

Figure S2. *Cont.*

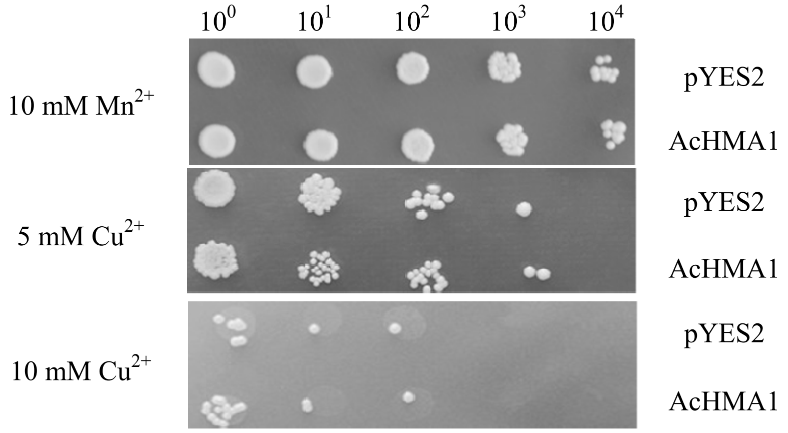

Table S1. Pair of primers of salt resistance-related genes and reference gene for quantitative RT-PCR.

Accession No.	Genes Description	Forward Primers	Reverse Primers
KF86390	heavy metal associated protein	5'-AGAGGTTACCAAGGGGTGGA-3'	5'-CAGTAATTGGGCGTTTCGGC-3'
KJ027085	EF1a	5'-CCCCAGTTCTCGACTGTCAC-3'	5'-TGGTGGGAACCATCTTCACG-3'
